# Neuroimaging evidence for central mechanisms of acupuncture in non-specific low back pain: a systematic review and meta-analysis

**DOI:** 10.3389/fmed.2025.1657241

**Published:** 2025-10-17

**Authors:** Frank Fan Huang, Jiajun Liu, Manqi Lu, Yixun Wu, Shanshan Zhen, Yihui Cai, Zhaoxue Liu, Mingwang Qiu, Wenwu Xiao, Yuxi Huang, Junquan Liang, Min Li, Zaigao Liu

**Affiliations:** ^1^Department of Rehabilitation Sciences, The Hong Kong Polytechnic University, Hong Kong, Hong Kong SAR, China; ^2^Tianjin Hospital, Tianjin University, Tianjin, China; ^3^Department of Rehabilitation Medicine, Shenzhen Hospital, Southern Medical University, Shenzhen, China; ^4^The Second School of Clinical Medicine, Guangzhou University of Chinese Medicine, Guangzhou, Guangdong, China; ^5^School of Exercise and Health, Shanghai University of Sport, Shanghai, China; ^6^Department of Rehabilitation Medicine, Affiliated Renhe Hospital of China Three Gorges University, Yichang, China; ^7^The Fourth School of Clinical Medicine, Guangzhou University of Chinese Medicine, Guangzhou, Guangdong, China; ^8^Shenzhen Bao’an Chinese Medicine Hospital, The Seventh Clinical Medical School of Guangzhou University of Chinese Medicine, Shenzhen, China; ^9^The Brain Cognition and Brain Disease Institute (BCBDI), Shenzhen Institute of Advanced Technology, Shenzhen, China; ^10^Chinese Academy of Sciences (CAS), Shenzhen-Hong Kong Institute of Brain Science-Shenzhen Fundamental Research Institutions, Shenzhen, China; ^11^University of Chinese Academy of Sciences, Beijing, China; ^12^Fifth School of Clinic Medicine, Guangzhou University of Chinese Medicine, Guangzhou, Guangdong, China; ^13^The Second Affiliated Hospital, School of Medicine, The Chinese University of Hong Kong, Shenzhen & Longgang District People’s Hospital of Shenzhen, Shenzhen, China

**Keywords:** acupuncture, non-specific low back pain, meta-analysis, functional magnetic resonance imaging, systematic reviews

## Abstract

**Objectives:**

Non-specific low back pain (NSLBP) is a prevalent disorder with significant global health impacts. This systematic review and meta-analysis assessed acupuncture’s clinical effectiveness for NSLBP and explored its brain mechanisms using fMRI.

**Methods:**

A comprehensive search of multiple databases (PubMed, Embase, Cochrane Library, Web of Science, Science Direct, China National Knowledge Infrastructure, Wanfang Data, Chinese Technical Periodicals Database, and Chinese Biomedical Literature Database) was conducted from inception to July 11th, 2024. We included randomized controlled trials (RCTs) or non-RCTs resting-state functional magnetic resonance imaging to observe the effect of acupuncture on NSLBP. GingerALE 3.0.2 was used as the meta-analysis tool, and meta-analysis was performed in the Montreal Neurological Institute coordinate space.

**Results:**

The review synthesized evidence from ten studies involving 358 participants. Subgroup analyses indicated that acupuncture significantly reduced pain scores compared to sham acupuncture in both acute NSLBP (WMD = −1.04, 95% CI: −1.72 to −0.36, *p* = 0.003) and chronic NSLBP (WMD = −0.78, 95% CI: −1.25 to −0.31, *p* < 0.001). Neuroimaging analyses revealed distinct brain activation patterns: acute NSLBP showed positive activation in the right sub-lobar insula, inferior parietal lobule, medial frontal gyrus, and cingulate gyrus, while chronic NSLBP demonstrated positive activation in bilateral sub-lobar insula and negative activation in motor and prefrontal regions.

**Conclusion:**

Acupuncture shows significant efficacy for NSLBP, modulating pain processing through the insula and limbic system. While these results suggest therapeutic potential for both acute and chronic NSLBP, higher-quality research is needed to validate these mechanisms.

**Systematic review registration:**

Prospero registration number: CRD42022342438, URL: https://www.crd.york.ac.uk/PROSPERO/view/CRD42022342438.

## 1 Introduction

Non-specific low back pain (NSLBP), a highly prevalent musculoskeletal disorder in adults, encompasses both nociceptive and neuropathic components that may radiate to the lower extremities, significantly impairing mobility and function (1). The classification of NSLBP falls into acute, subacute, and chronic categories (2, 3). According to the 2021 Global Burden of Disease Study, NSLBP ranks among the top 10 causes of long-term disability in 188 countries (2, 4). The global prevalence of lower back pain is estimated at 18.3%, with higher rates observed among women and in high-income countries (5). Financially, this condition imposes a heavy burden, costing the UK approximately £2.8 billion annually, Australia over $4.8 billion, and the US more than $100 billion (6).

Given its impact, effective treatments for NSLBP are critical for global health. Opioids are frequently prescribed for chronic NSLBP but raise concerns about addiction and risks (7), contributing to a drug abuse crisis and fueling demand for non-opioid alternatives (8). Increasingly, research has pointed to non-pharmacological approaches as safe and effective alternatives for managing NSLBP (9–13), and the effectiveness of acupuncture in pain relief has been demonstrated in numerous studies (14–16). It is also strongly advised to utilize acupuncture for treatment in the American College of Physicians guidelines for treating chronic NSLBP (13).

Regular MRI is used to visualize structural abnormalities such as disc herniations, spinal stenosis, or cancer. Brain imaging studies reveal stage-specific alterations in NSLBP. SPECT imaging and statistical analyses have demonstrated different alterations in brain blood flow among patients with acute and chronic NSLBP (17). In chronic cases, enhanced connectivity within the frontoparietal network (FPN), somatomotor network (SMN), and thalamus (18). This increased connectivity represents neurophysiological changes associated with the chronic phase of the condition. Given these altered connectivity patterns of different phrases of NSLBP, acupuncture has been explored as a potential neuromodulatory intervention. As for mechanism, acupuncture appears to influence several brain networks involved in pain, emotion, and memory, such as the sensorimotor network, the default mode network (DMN), and the limbic system (19). However, the exact neurophysiological mechanisms remain unclear due to acupuncture’s engagement of multiple neural circuits (20), highlighting the need for further research to clarify its role across NSLBP phases.

Since the mid-1990s, functional magnetic resonance imaging (fMRI) has been used to observe the human brain’s response to acupuncture stimulation (21). As an imaging method, fMRI reveals time-varying changes in brain metabolism, offering researchers precise insights into the anatomical and physiological functions associated with acupuncture. These findings suggest that acupuncture’s mechanism is mediated through the central nervous system (19). Therefore, fMRI is a critical tool for investigating how acupuncture exerts its therapeutic effects at the neurophysiological level. Acupuncture’s analgesic effects are mediated by neurotransmitters, signaling pathways, and immunological responses, which in turn influence neural activity in specific brain regions (22). Previous study discovered that following acupuncture therapy, neural activation increased in the sensorimotor network, periaqueductal grey, and nucleus accumbens, while the DMN showed decreased activation (23). Moreover, there were common patterns of activation in the sensorimotor cortical network and deactivation in the limbic paralimbic neocortical network after acupuncture stimulation (24). These effects were also observed in participants with NSLBP, where acupuncture improved aberrant brain structure and functional activity, primarily through the pain matrix, DMN, salience network, and descending pain modulatory system (25). In summary, acupuncture’s ability to modulate brain networks and neurotransmitter activity contributes to its therapeutic effects on pain.

Several reviews have summarized the mechanisms underlying this treatment using magnetic resonance imaging to explore its effects on NSLBP (26–29). Yet, these analyses did not differentiate NSLBP by duration, limiting understanding of phase-specific analgesic mechanisms. Addressing this gap, our meta-analysis categorizes NSLBP into acute, subacute, and chronic phases to examine pain scales and brain function following acupuncture. By focusing on duration-specific cohorts, our study aims to elucidate neural substrates of acupuncture analgesia, informing clinical decisions and guiding future research directions.

## 2 Methods

### 2.1 Data and methods

The protocol of this study was registered at PROSPERO (http://www.crd.york.ac.uk/PROSPERO) (registration number: CRD42022342438). A systematic review was conducted in accordance with the Preferred Reporting Items for Systematic Reviews and Meta-Analyses guidelines (PRISMA guidelines) and neuroimaging guidelines for meta-analyses (30).

### 2.2 Literature retrieval

A systematic search strategy was conducted in PubMed, Embase, Cochrane Library, Web of Science, Science Direct, Medline, China National Knowledge Infrastructure, Wanfang Data Knowledge Service Platform, Chinese Technical Periodicals Database and Chinese Biomedical Literature Database from inception to July 11th, 2025. Additionally, forward citation tracking were identified by manually searching the included studies. The electronic search procedures are presented in Supplementary materials.

### 2.3 Inclusion/exclusion criteria

Studies were included based on the following criteria: (1) Randomized controlled trial (RCT) or non-RCT conducted in patients with acute, subacute or chronic NSLBP. Acute back pain is defined as lasting less than 4 weeks, subacute back pain lasts 4 to 12 weeks, and chronic back pain lasts more than 12 weeks (17); (2) fMRI study; (3) acupuncture as the intervention; (4) other therapies including conventional rehabilitation or sham acupuncture as the control group; (5) study setting in clinic, community, hospital, or laboratory; (5) presenting the results in Talairach or Montreal Neurological Institute (MNI) coordinates.

The exclusion criteria were as follows: (1) abstracts, case reports, commentaries, conference papers, cohort studies, cross-sectional studies, descriptive studies, editorials or expert opinions,or letters; (2) animal trial; (3) no extractable data available; (4) not published in English or Chinese.

### 2.4 Data extraction

For the data extraction, the base information of the author, country, condition, sample size of trial groups and control groups, participant characteristics, duration of NSLBP, outcomes, interventions, methodological quality assessment tool and main conclusions were extracted according to the PRISMA flowchart (31).

Firstly, the clinical outcome measures included assessing pain intensity and functional status. Pain intensity is primarily assessed using the visual analogue scale. Functional status can be assessed through self-reported questionnaires measuring disabilities for functional evaluation (e.g., Roland Disability Questionnaire for Sciatica, World Health Organization Quality of Life in the Brief Edition). Secondly, the outcome measures also included brain imaging. For brain imaging data, the brain-related data including magnetic resonance imaging model, field strength (Tesla), head coil, fMRI acquisition parameters [repetition time (TR): 2000–3,000 ms; echo time (TE): 30–40 ms; voxel size: 2.6 × 2.6 × 3.0 mm^3^ to 3.4 × 3.4 × 4.0 mm^3^], software used for analysis (e.g., SPM, FreeSurfer), coordinate space (MNI or Talairach), smoothing kernel (full-width at half-maximum: 5–8 mm), type I error correction, and functional imaging feature were extracted. Preprocessing steps of fMRI data in included studies consistently included: (1) motion correction; (2) slice-timing correction; (3) normalization to MNI space; (4) spatial smoothing. The coordinates and information for each study were manually extracted by two researchers (F. H. and M. Q. L.) and independently checked for accuracy by the other author (J. J. L.).

### 2.5 Methodological quality assessment and level of evidence

We employed Risk of Bias 22 and Risk Of Bias In Non-randomized Studies-of Interventions tools to evaluate the risk of bias in the included RCTs and non-RCTs, respectively (32, 33). For RCTs, the assessment focused on several bias sources: bias arising from the randomization process, bias due to deviations from intended interventions, bias due to missing outcome data, bias in outcome measurement, bias in the selection of reported results, and overall risk of bias. Based on these criteria, the risk of bias in RCT studies was categorized as low risk, some concerns, or high risk. In the case of non-RCTs, the assessment considered factors such as bias due to confounding, bias in participant selection, bias in the classification of interventions, bias due to deviations from intended interventions, bias due to missing data, bias in outcome measurement, bias in the selection of reported results, and overall risk of bias. According to these criteria, the risk of bias in non-RCT studies was classified as low, moderate, serious, critical, or no information.

### 2.6 Data analysis

Stata 12.0 software (Stata Corp, College Station, TX, USA) was used for clinical data meta-analysis. Dichotomous outcomes were reported using risk ratios with corresponding 95% confidence intervals (CIs). Continuous outcomes were presented as weighted mean differences (WMDs) with 95% CIs or standardized mean differences. A fixed-effects model was employed when the *I^2^* statistic was below 50% Otherwise, a random-effects model was utilized. Subgroup analysis was also conducted. And the level of evidence was used by The Grading of Recommendations, Assessment, Development and Evaluation (GRADE) approach (34).

GingerALE 3.0.2 (http://www.brainmap.org/ale/) is a tool used for neuroimaging meta-analyses, which converts all reported coordinates into MNI space via the icbm2tal transformation. Anatomical structures were identified within the software, with parameters set at *p* ≤ 0.001 (cluster-level family-wise error correction = 0.001) (35–38). Mango 4.0.2 (Research Imaging Institute, UTHSCSA) was used for visualization, mapping the three-dimensional ALE results onto the MNI standard template to facilitate precise localization of brain regions.

### 2.7 Activation likelihood estimation procedure

In ALE analysis, activation hotspots found in existing research were viewed as probability patterns centred on the reported coordinates. For each voxel in a standard space, activation probabilities were determined to create ALE maps that focus on particular contrasts. To assess the trustworthiness of these ALE maps, null distributions were formed by examining how ALE values were distributed across separate studies (36). This approach was somewhat like performing permutation tests on individual voxels from different experiments. The influence of each study in the analysis was adjusted based on its sample size, and each study is considered to contribute to random effects (35).

### 2.8 Calculation of frequency about brain regions modulated by acupuncture

To summarize and visualize the frequency of brain regions modulated by acupuncture in acute and chronic LBP, we utilized Excel for data analysis. The frequencies of involvement for various brain regions were calculated and plotted using Excel’s graphing capabilities. This allowed us to effectively illustrate the distribution of modulated regions in both acute and chronic LBP.

### 2.9 Sensitivity analysis

We performed sensitivity analyses to assess the robustness of ALE meta-analysis results based on previous article (37). Studies with a total sample size of <20 were excluded to address potential small sample bias.

## 3 Results

### 3.1 Study search results

A total of 1,020 articles were identified through PubMed, Embase, Cochrane Library, Web of Science, Science Direct, China National Knowledge Infrastructure, Wanfang Data Knowledge Service Platform, Chinese Technical Periodicals database and Chinese Biomedical Literature Database. After removing duplicates, trials for which no full-text was available, and screening titles and abstracted, a total of ten studies were included for further evaluation (8, 39–47).

### 3.2 Characteristics of the included studies

**Figure 1:**
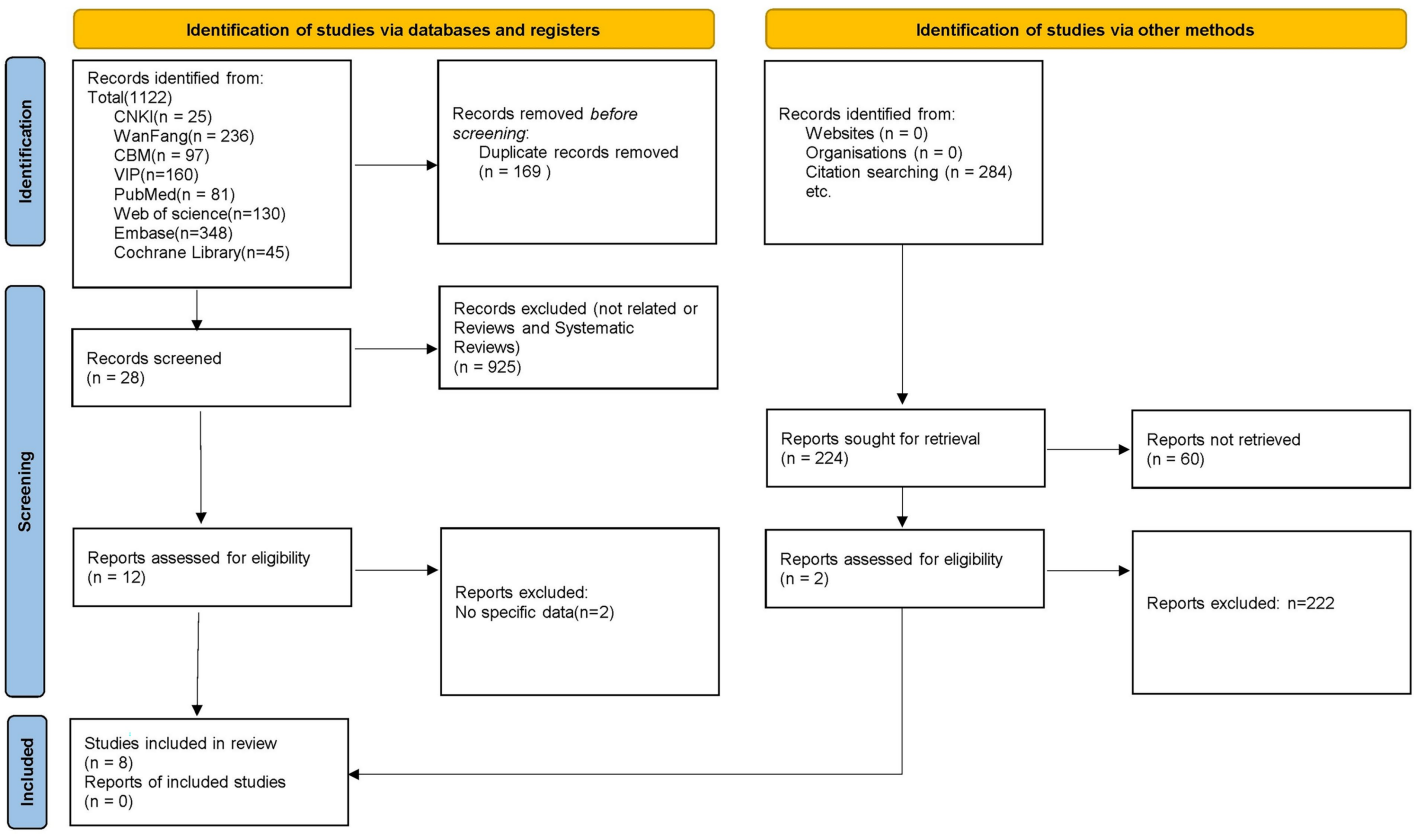
Flowchart of the study selection process.

A total of 358 participants were involved in ten articles (Cohen’s kappa = 0.85) (8, 39–47). Table 1 shows the characteristics of the included studies. Among these ten articles, three are about acute non-specific low back pain (ANSLBP) (39, 40, 42) and seven are about chronic non-specific low back pain (CNSLBP) (8, 41, 43–47). Among the ten studies included, eight (8, 37, 39, 40, 42, 43, 45, 46) were divided into an acupuncture group and a control group, of which three (39, 40, 42) analyzed the effect of acupuncture on ANSLBP, and the other five (8, 43, 45–47) analyzed the effect of acupuncture on CNSLBP. No subacute NSLBP articles were included. The other two studies (41, 44) had no control group. The selection process is shown in Figure 1.

**Table 1:** Characteristics of the included studies.

Study	Sample number	Phrase	Location	Design	Treatment duration	Age	Treatment group	Control group	Manipulation modality	Imaging type	MRI parameter	Clinical variables	Adverse events	Outcomes
Ziping (40)	Sum = 15	acute	hospital	RCT	16 min	25.7 ± 2.3	acupuncture	sham acupuncture	MA	RS-fMRI	3 T, MNI	1. Vas score 2. ASS	None	Brain activation
Shi et al. (42)	Sum = 28	acute	NA	RCT	36 min	22–30	acupuncture	sham acupuncture	EA	RS-fMRI	3 T, MNI	1. Vas score	None	ReHo, brain activation
Makary, 2018 (46)	TG = 28 CG = 19	chronic	laboratory	RCT	25 min	38.4 ± 12.7	acupuncture	sham acupuncture	MA	RS-fMRI	3 T, MNI	1. Vas score	None	ROI
Lee, 2019 (43)	TG = 25 CG = 18	chronic	NA	RCT	25 min	38.4 ± 12.7	acupuncture	sham acupuncture	MA	RS-fMRI	3 T, MNI	1. Vas score	None	ROI, FC
Tu, 2019 (45)	TG = 24 CG = 26	chronic	NA	RCT	25 min, 1 or 2 times/week, 2 weeks	26–54	acupuncture	sham acupuncture	MA	TS-fMRI	3 T, MNI	1. Vas score 2. BDI 3. ERS	None	rsFC
Yu, 2020 (8)	TG = 24 CG = 26	chronic	hospital	RCT	25 min, 1 or 2 times/week, 2 weeks	18–60	acupuncture	sham acupuncture	MA	TS-fMRI	3 T, MNI	1. Vas score 2. BDI 3. Bothersomeness scale	None	rsFC
Kim, 2020 (57)	TG = 55 (TG1 = 18, TG2 = 18, TG3 = 19) CG = 23	chronic	hospital	RCT	20 min, 1 or 2 times/week, 4 weeks	41.2 ± 12.0	acupuncture	None	MA	TS-fMRI	3 T, MNI	1. Vas score 2.2PDT	None	ROI
Xiang, 2021 (11)	Sum = 19	chronic	laboratory	Non-RCT	15 min	46.61 ± 7.35	ankle acupuncture	sham acupuncture	MA	RS-fMRI	3 T, MNI	1. Vas score	None	ALFF

BDI, Beck Depression Inventory; TG, treatment group; CG, control group; EA, electroacupuncture; MA, manual acupuncture; ERS, expectations for relief scale; JOA, Japanese Orthopaedic Association scores; NA, not available; Sham, sham acupuncture; VAS, visual analogue scale; Verum, verum acupuncture; 2 PDT, two-point discrimination threshold; ASS, Acupuncture Sensation Scale; rs-FC, resting-state functional connectivity/FC; ALFF, Amplitude of Low-Frequency Fluctuations; ICC, Intrinsic Connectivity Contrast; Reho, Regional Homogeneity; ROI, Regions of Interest.

### 3.3 Quality assessment of the included trials

In eight RCTs (8, 39, 40, 42, 43, 45–47), one study was rated as “low” overall risks of bias (8), while seven studies were rated as “some concerns” of overall risks of bias due to concerns about the randomization process (39, 40, 42, 43, 45–47). In two non-RCTs (41, 44), all two studies were rated as “low” overall risks of bias (Supplementary Tables 1, 2). According to the GRADE approach, the quality of evidence and the strength of recommendations were rated as “very low” (Supplementary Table 3).

### 3.4 Meta-analysis results of pain-related scales

**Figure 2:**
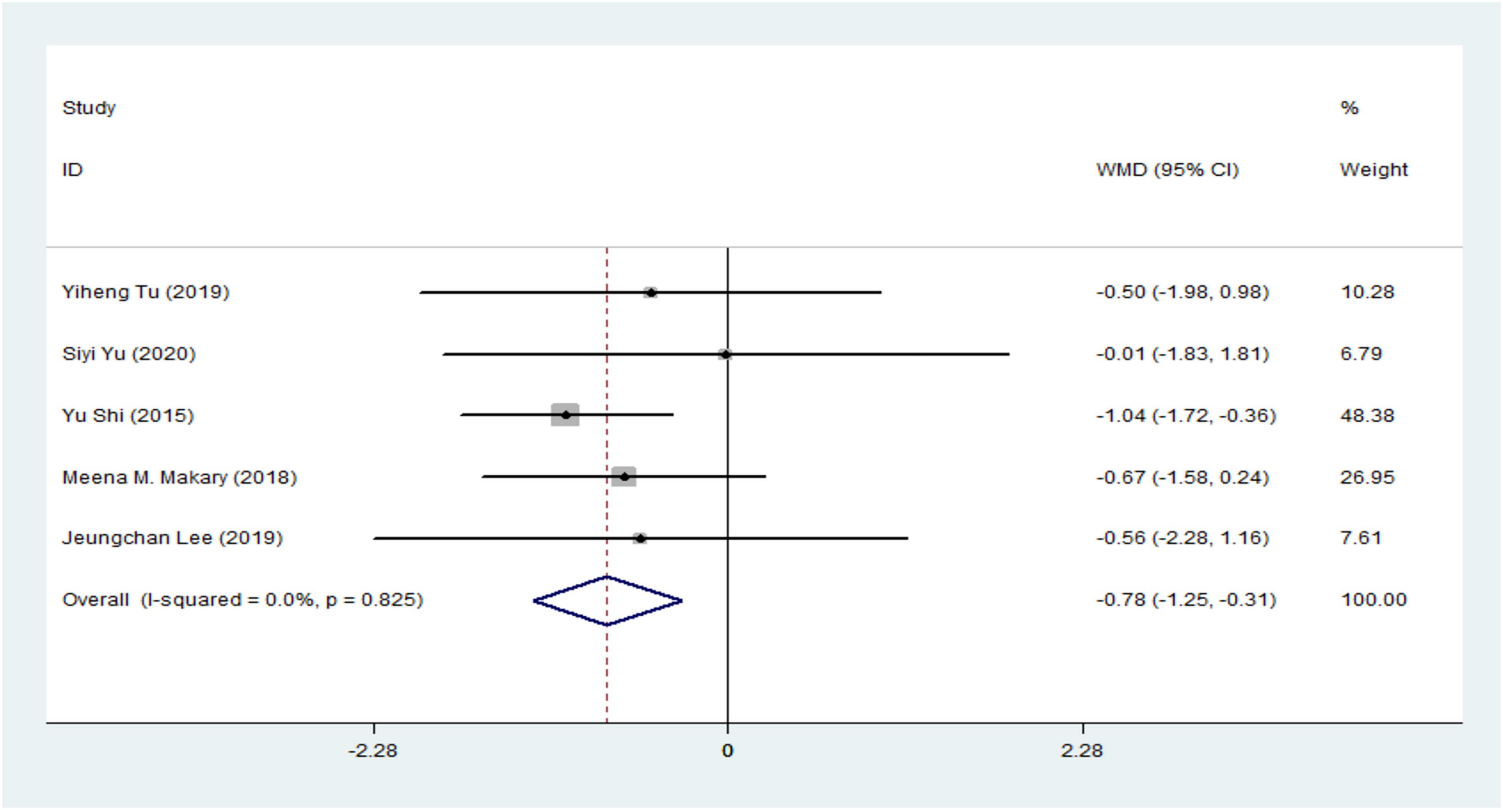
Forest plot of the sum total.

Based on the pooled results from five RCTs (Supplementary Figure 1) (8, 43, 45–47), the acupuncture group showed significantly lower pain-related scores of VAS compared with the sham acupuncture group, as illustrated in Figure 2 (5 trials: WMD = −0.78, 95% CI: −1.25 to −0.31, *p* < 0.001), with no heterogeneity (*I*^2^ = 0%, *p* = 0.825) For subgroup analysis, one RCT demonstrated that acupuncture was significantly more effective than sham acupuncture for treating ANSLBP (WMD = −1.04, 95% CI: −1.72 to −0.36, *p* = 0.003) (42). However, four RCTs on CNSLBP showed no significant difference between real and sham acupuncture (Figure 1) (WMD = −0.53, 95% CI: −1.19 to 0.13, *p* = 0.113) (8, 43, 45–47), with no heterogeneity (*I*^2^ = 0%, *p* = 0.938).

#### 3.4.1 Neuroimaging findings after acupuncture for ANSLBP

**Figure 3:**
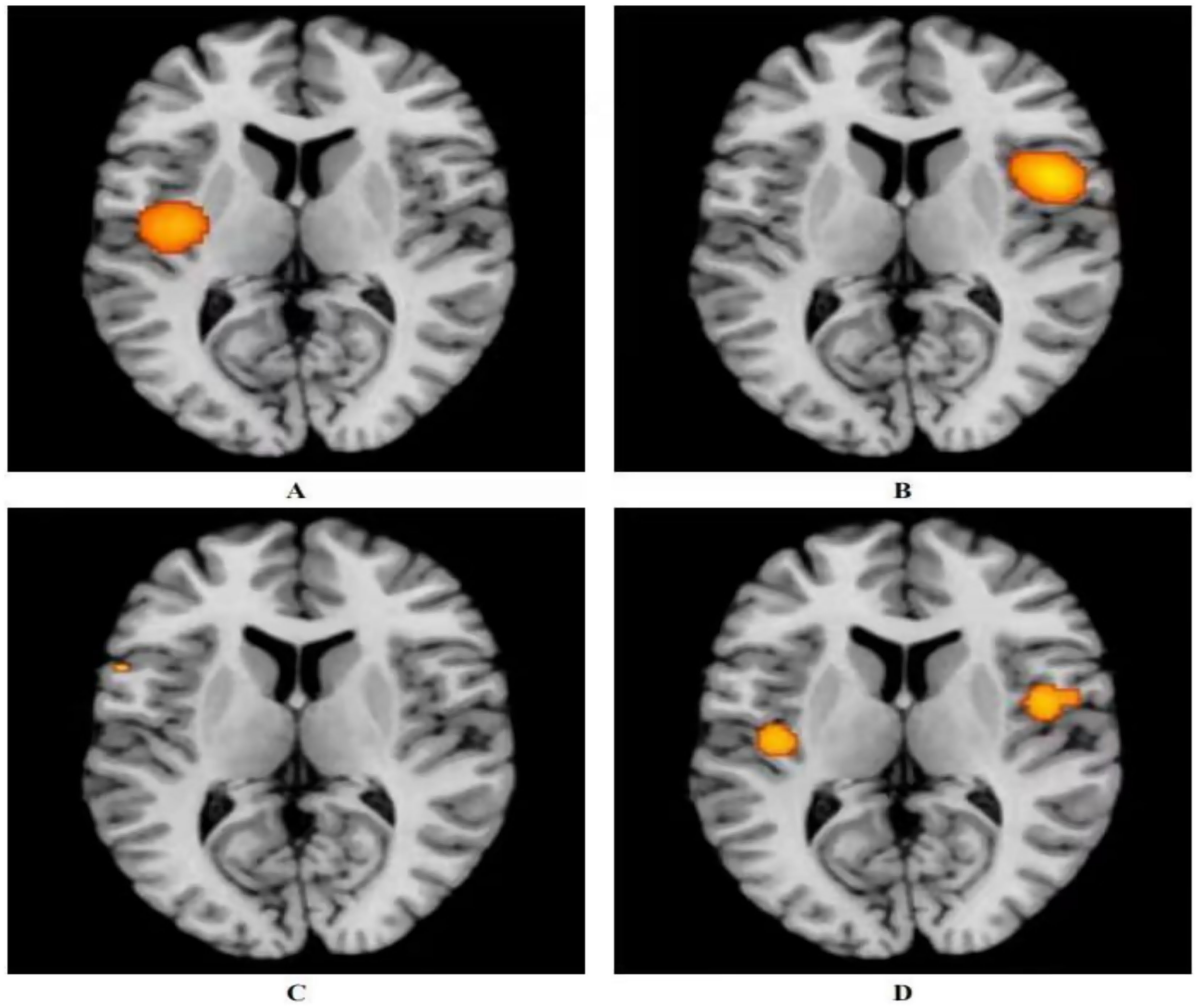
Activation of fMRI signals in cortical and subcortical structures in the acupuncture group. **(A)** Positive activation of brain regions after acupuncture with ANSLBP; **(B)** Negative activation of brain regions after acupuncture with ANSLBP; **(C)** Positive activation of brain regions after acupuncture with CNSLBP; **(D)** Negative activation of brain regions after acupuncture with CNSLBP.

Three studies utilized acupuncture for the treatment of ANSLBP (39, 40, 42). Following the ALE meta-analysis of these articles, the results identified four clusters of positive activation and seven clusters of negative activation (Figure 3).

Four clusters of positive activation were identified. The first cluster was located in the right cerebrum, specifically in the sub-lobar insula (Brodmann area 13), centered at coordinates *x* = 50, *y* = 6, *z* = 12 (ALE = 0.0022; *p* < 0.001; *Z* = 5.20). The second cluster was found in the right cerebrum, in the inferior parietal lobule (Brodmann area 40), centered at *x* = 62, *y* = −26, *z* = 34 (ALE = 0.0023; *p* < 0.001; *Z* = 5.39). The third cluster was situated in the right cerebrum, in the medial frontal gyrus (MFG) (Brodmann area 6), centered at *x* = 12, *y* = 0, *z* = 60 (ALE = 0.0019; *p* < 0.001; *Z* = 4.85). The final cluster was located in the right cerebrum, in the cingulate gyrus (Brodmann area 31), centered at *x* = 18, *y* = −24, *z* = 39 (ALE = 0.0019; *p* < 0.001; *Z* = 4.84) (Figure 3A; Supplementary Table 4).

Seven clusters of negative activation were identified. The first cluster was located in the left cerebrum, specifically in the sub-lobar insula (Brodmann area 13), centered at coordinates *x* = −41, *y* = −13, *z* = 15 (ALE = 0.0027; *p* < 0.001; *Z* = 6.36). The second cluster was found in the left cerebrum, in the cingulate gyrus (Brodmann area 32), centered at *x* = 0, *y* = 33, *z* = 21 (ALE = 0.0028; *p* < 0.001; *Z* = 7.03). The third cluster was situated in the left cerebrum, in the pulvinar of the thalamus, centered at *x* = −4, *y* = −30, *z* = −2 (ALE = 0.0019; *p* < 0.001; *Z* = 4.58). The fourth cluster was located in the right cerebrum, in the parahippocampal gyrus (Brodmann area 35), centered at *x* = 24, *y* = −27, *z* = −18 (ALE = 0.0019; *p* < 0.001; *Z* = 4.61). The fifth cluster was found in the right cerebrum, in the MFG (Brodmann area 8), centered at *x* = 14, *y* = 33, *z* = 44 (ALE = 0.0019; *p* < 0.001; *Z* = 4.57). The sixth cluster was located in the right cerebrum, in the angular gyrus (Brodmann area 39), centered at *x* = 54, *y* = −60, *z* = 39 (ALE = 0.0019; *p* < 0.001; *Z* = 4.61). The final cluster was situated in the left cerebrum, in the superior frontal gyrus (Brodmann area 6), centered at *x* = −14, *y* = 34, *z* = 52 (ALE = 0.0019; *p* < 0.001; *Z* = 4.57) (Figure 3B; Supplementary Table 5).

#### 3.4.2 Neuroimaging findings after acupuncture for CNSLBP

Seven studies utilized acupuncture as a treatment for CNSLBP (8, 41, 43–47). Analyzing the related articles revealed two clusters of positive activation and two clusters of negative activation (Figure 3).

The two positive activation clusters identified were as follows: one was located in the right cerebrum, specifically in the sub-lobar insula (Brodmann area 13), centered at coordinates *x* = 46, *y* = −2, *z* = 2 (ALE = 0.0019; *p* < 0.001; *Z* = 4.65). The other was found in the left cerebrum, also in the sub-lobar insula (Brodmann area 13), centered at *x* = −42, *y* = −16, *z* = 2 (ALE = 0.0015; *p* < 0.001; *Z* = 3.93) (Figure 3C; Supplementary Table 6).

The two negative activation clusters were located as follows: one was situated in the left cerebrum, in the precentral gyrus (Brodmann area 44), centered at *x* = −56, *y* = 12, *z* = 6 (ALE = 0.00095; *p* < 0.001; *Z* = 3.41). The other was located in the right cerebrum, in the middle frontal gyrus (Brodmann area 8), centered at *x* = 33, *y* = 40, *z* = 42 (ALE = 0.00095; *p* < 0.001; *Z* = 3.41) (Figure 3D; Supplementary Table 7).

#### 3.4.3 Neuroimaging results after the control group’s treatment on ANSLBP

**Figure 4:**
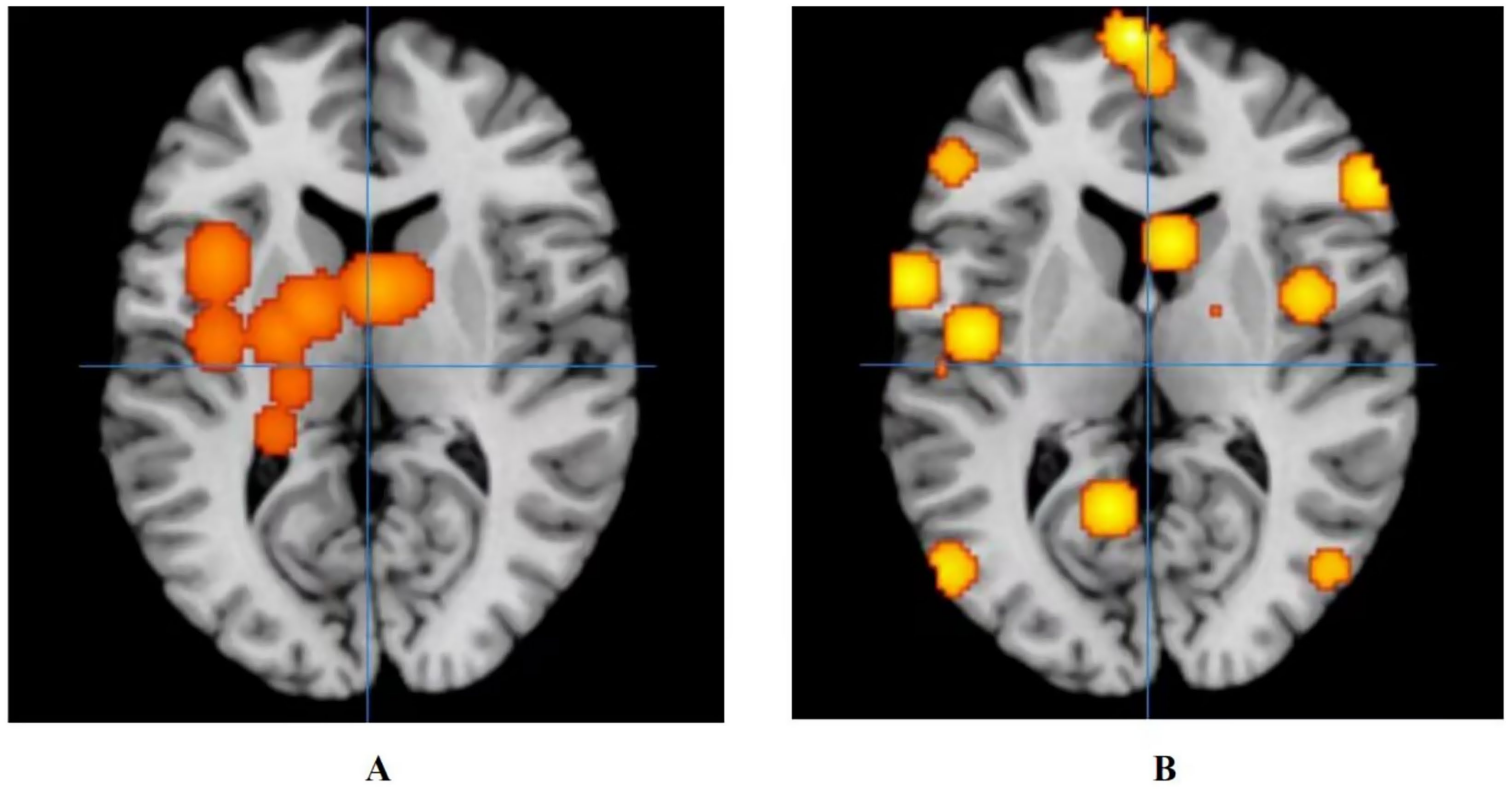
Activation of fMRI signals in cortical and subcortical structures in the control group. **(A)** Activation of brain regions in patients with ANSLBP after receiving treatment in control group; **(B)** Activation of brain regions in patients with CNSLBP after receiving treatment in control group.

The results showed that the control group’s treatment for ANSLBP activated over eight clusters, predominantly located in the sub-lobar regions of the left cerebrum, including the insula, lentiform nucleus, thalamus, caudate, and hippocampus. Only the caudate in the right cerebrum showed activation. It was clear that most of the activated clusters were in the sub-lobar area. Additionally, the ALE values ranged from 0.0009 to 0.001, with the insula in the left cerebrum displaying the highest activation, specifically centered at *x* = −40, *y* = 6, *z* = 18 (ALE = 0.0014, *p* < 0.001, *Z* = 4.05) (Figure 4A). In summary, the results indicate that significant brain activation occurs primarily in the sub-lobar region, with the left insula showing the highest activation, which may be central to the neurophysiological response to ANSLBP treatment.

#### 3.4.4 Neuroimaging results after the control group’s treatment on CNSLBP

Long-term control group’s regulates wider areas, such as the limbic lobe (Brodmann area 40; peak MNI coordinates: −50, −28, 52; peak SDM-Z (Seed-based d Mapping): 3.597; *p* < 0.001), the parietal lobe (Brodmann area 36; peak MNI coordinates: −50, −34, −16; peak SDM-Z: 3.597; *p* < 0.001), the frontal lobe (Brodmann area 6; peak MNI coordinates: 44, 6, 30; peak SDM-Z: 3.597; *p* < 0.001), the temporal lobe (Brodmann area 22; peak MNI coordinates: −66, −42, 18; peak SDM-Z: 3.597; *p <* 0.001), the sub-lobar (Caudate Body; peak MNI coordinates: 18, −4, 16; peak SDM-Z: 3.597; *p* < 0.001), and the occipital lobe (Brodmann area 37; peak MNI coordinates: −52, −72, 4; peak SDM-Z: 3.597; *p* < 0.001) (Figure 4B).

### 3.5 Sensitivity analysis

Two studies with small sample sizes were excluded: Ziping (40; total *n* = 15) and Xiang et al. (11) (total *n* = 19). After exclusion, only one study on ANSLBP remained, while all CNSLBP studies met the sample size criterion (n ≥ 20). Consistent with the initial analysis, the key neuroimaging findings remained unchanged. Acupuncture treatment on ANSLBP showed positive activation in the right sub-lobar insula, inferior parietal lobule, medial frontal gyrus, and cingulate gyrus; CNSLBP demonstrated positive activation in the bilateral sub-lobar insula and negative activation in the motor and prefrontal regions. Due to low heterogeneity (*I*^2^ = 0%) and consistent study inclusion, no re-calculation of ALE statistics was required, confirming the stability of the primary results.

### 3.6 Frequency of brain regions modulated by acupuncture in acute and chronic LBP

By combining these findings, we summarized and visualized the frequency of brain regions modulated by acupuncture in acute and chronic LBP (Supplementary Figure 2). As shown in the Supplementary Figure 2, in ANSLBP, the most frequently involved regions were the insula and lentiform nucleus (approximately 75%), followed by the thalamus, caudate, and hippocampus (around 25%). In CNSLBP, the most frequently involved regions were the middle frontal gyrus (>50%) and precentral gyrus (approximately 45%), followed by the parahippocampal gyrus, anterior cingulate cortex, superior parietal lobule, and inferior parietal lobule (about 20–30%).

### 3.7 Adverse events

As shown in Table 1, none of the RCTs or non-RCTs reported adverse events.

## 4 Discussion

This study compares brain changes following acupuncture treatment of acute and chronic NSLBP using pain-related scales and resting-state fMRI. The research results indicated that acupuncture has demonstrable clinical efficacy for treating NSLBP. Through a meta-analysis of all eligible articles, three studies used acupuncture for ANSLBP (39, 40, 42), with brain activation mainly in the bilateral limbic lobe and right inferior lobe. In the seven studies of acupuncture treatment of CNSLBP (8, 41, 43–47), we identified four clusters of activation, including the sub-lobar insula, precentral gyrus on the left side, and the sub-lobar insula and middle frontal gyrus on the right side.

### 4.1 Pain-related outcomes analysis of acupuncture for NSLBP

Acupuncture is currently recognized as an effective treatment for spinal-related diseases. In recent years, RCTs have demonstrated their role in treating degenerative diseases, chronic pain, and acute pain (48, 49). Chen et al. found that patients with chronic low back pain exhibit widespread alterations in brain regions related to pain perception and modulation, including the left inferior temporal gyrus, bilateral postcentral gyrus, superior and middle frontal gyri, thalamus, and occipital cortex. Notably, acupuncture appears to modulate functional activity in several of these pathological areas (50). Specifically, increased cerebral blood flow has been observed in the right postcentral gyrus and superior parietal lobule (regions implicated in somatosensory processing and sensorimotor integration), while in the bilateral occipital cortex and posterior cingulate gyrus is reduced (51).

In addition to targeted brain modulation by acupuncture, it is useful to compare its effects with those of other non-acupuncture treatments for NSLBP. Acupuncture has been shown to restore altered DMN connectivity, particularly in the dorsolateral and medial prefrontal cortices, anterior cingulate, and precuneus, with these changes correlating with pain relief (52). Similarly, physical or manual therapies, such as spinal manipulative therapy (SMT), modulate DMN regions including the right parahippocampal gyrus, posterior cingulate cortex, and precuneus, indicating altered intrinsic connectivity related to pain processing (53). Cognitive Behavioral Therapy (CBT) engages cognitive control and emotional regulation networks, with magnetoencephalography studies showing normalization of activity in the right inferior frontal gyrus and dorsolateral prefrontal cortex, correlating with pain reduction (54). Structural MRI further reveals increased gray matter in the dorsolateral prefrontal and posterior parietal cortices after CBT, associated with decreased catastrophic thinking (55, 56).

However, among the articles we included, only three addressed acute pain. Clinically, acupuncture is sometimes used for acute low back pain in emergency settings of traditional Chinese medicine clinics, making research challenging due to low follow-up rates. Conversely, patient compliance is higher for chronic back pain, resulting in more reliable therapeutic outcomes in the included articles. Although our meta-analysis did not demonstrate positive results without grouping, this might be due to the efficacy of the control group (i.e., open-label studies, lack of blinding). Such variability in control group selection may affect meta-analysis results. Nonetheless, the treatment effectiveness of acupuncture for chronic NSLBP remains significantly different when compared with healthy controls in our study, confirming its clinical relevance and ongoing research importance in traditional Chinese medicine.

### 4.2 Neuroimaging analysis of acupuncture for NSLBP

According to our findings, both acute and CNSLBP activate the right insula following acupuncture, a region crucial for integrating sensory processing and cognitive regulatory systems (45). Activation of the insula observed in the acupuncture group was accompanied by significant reductions in VAS scores, suggesting that modulation of this key pain-processing region may underlie acupuncture’s clinically meaningful analgesic effects. Research suggests that the anterior insula plays a key role in the salience network, responsible for identifying and filtering salient stimuli, particularly during exposure to unpleasant stimuli (57). Acupuncture has been shown to reduce cross-network functional connectivity between the insula and the DMN, and this reduction correlates with the degree of clinical pain alleviation (47). These findings suggest that right insula activation is critical to acupuncture’s analgesic effects for both acute and chronic NSLBP.

Beyond the insula, the limbic lobe, located at the cerebral cortex’s periphery, also plays a significant role in pain processing (58). Regions such as the amygdala, orbitofrontal cortex, hippocampus, and cingulate cortex form part of this network (59). The anterior cingulate cortex (ACC), in particular, is involved in emotion and behavior regulation (60). Acupuncture somatosensory afference can transmit tactile information from the spinal cord to the thalamus, periaqueductal grey, and reticular formation, subsequently affecting the ACC, insula, and sensory cortices (61). Activation in the ACC, especially in its dorsal sub-region, has been linked to acute pain stimulation, suggesting that the ACC’s activation in this study may correspond to acupuncture’s pain-relieving effects. (dACC, BA 24) (62). Therefore, the insula, ACC, and other limbic structures appear to mediate acupuncture’s analgesic effects in both acute and chronic NSLBP.

We also summarized and visualized the frequency of brain regions modulated by acupuncture in acute and chronic LBP. In ANSLBP, the insula and lentiform nucleu, followed by the thalamus, caudate, and hippocampus areas are associated with pain perception, emotional processing, and pain-related memory (63, 64). In CNSLBP, the middle frontal gyrus, precentral gyrus, followed by the parahippocampal gyrus, anterior cingulate cortex, superior parietal lobule, and inferior parietal lobule are more closely related to motor planning (65), execution (66), emotional regulation, attention control, and the persistence of chronic pain. Therefore, acute low back pain is more associated with nociceptive processing and emotion/memory circuits (insula–basal ganglia–limbic system), whereas chronic low back pain is more related to higher-order cognitive and motor control networks (frontal–parietal).

### 4.3 Analysis of the current neuroimaging results in the control group

The activated brain regions in the acupuncture group were primarily located in the bilateral limbic lobe and right inferior lobe, while the control group for ANSLBP primarily exhibited changes in the limbic system, basal ganglia, and thalamus (39, 40, 42). In contrast, the control group for CNSLBP showed a wider range of activation, including the frontal, temporal, sub-lobar, and occipital lobes (8, 41, 43–46). The lack of blinding in the control group, combined with open-label placebos, likely amplified this reward effect and further alleviated pain (67). These regions, particularly in the somatosensory cortex and pain conduction system, are crucial components of the central nervous system that regulate pain (68–70). Interestingly, despite the lack of a correlation analysis between ANSLBP and CNSLBP after sham acupuncture, there appears to be a similar modulation pattern in the limbic system across both conditions. This observation suggests that the limbic system may play a significant role in alleviating NSLBP. Comprising cortical and subcortical structures such as the prefrontal cortex (PFC), cingulate gyrus, hippocampus, and amygdala, the limbic system integrates sensory input from the environment to regulate emotional, autonomic, motor, and cognitive responses essential for survival (71–74). Previous research highlights the involvement of the reward system in acupuncture’s effects (75–77), particularly the PFC’s role in self-regulation and pain relief (78, 79). Taken together, the sensory stimuli received by the control group may convey positive reinforcement through the limbic system, particularly the PFC, contributing to pain reduction.

Our findings and previous literature indicate that sham acupuncture often induces neural activations in brain regions associated with attention, expectation, and pain modulation, reflecting placebo-related and nonspecific neural responses rather than acupuncture-specific effects (25, 80). This overlap complicates the interpretation of neuroimaging results and underscores the necessity of cautious attribution of brain activity solely to acupuncture. Future studies should further delineate these mechanisms to improve the specificity of acupuncture-related neurobiological findings.

### 4.4 Advantages and limitations

Neuroimaging results on the effects of acupuncture on NSLBP have been elusive, particularly due to the varied causes of the condition and differences in pain types (81). The duration of NSLBP may also significantly affect experimental outcomes, adding complexity to studies. Besides, Variability in acupuncture protocols (e.g., needle retention: 15–36 min; point selection) may confound neuroimaging effects. Future trials should adhere to the standards for reporting interventions in clinical trials of acupuncture guidelines. Additionally, the definition of chronic pain remains unclear (82, 83), which may lead to inaccuracies in clinical diagnosis and complicate research on brain function changes associated with chronic pain. One limitation of earlier studies is their failure to differentiate between acute and chronic NSLBP (84). Furthermore, pooling data from studies with different designs in meta-analyses can introduce heterogeneity and bias (85). In addition, the limited number of included studies in certain subgroup analyses (e.g., only three ANSLBP studies) restricts the statistical power of our findings, which should be considered when interpreting the results. Accordingly, further research with larger sample sizes is needed to yield more robust evidence. Besides, most included studies were rated as “very low” quality according to the GRADE approach, which weakens the strength of our conclusions. Future fMRI research on acupuncture should focus on methodological enhancements, such as rigorous randomization, appropriate blinding, and adequate sample size calculation, to improve evidence reliability. Despite the low quality of the study design, all MRI scans were conducted using 3 T machines, guaranteeing reliable imaging findings. Our study, however, addresses these limitations by distinguishing between acute and chronic NSLBP, allowing for a clearer comparison of acupuncture’s effects on brain function changes. This distinction helps resolve inconsistencies in prior research. Our pooled results offer a comprehensive overview of the post-acupuncture effects on clinical outcomes and brain activation in patients with NSLBP, providing valuable insights for both clinicians and researchers (85).

## 5 Conclusion

Acupuncture has shown considerable clinical efficacy in alleviating pain for patients with NSLBP, with key brain regions such as the sub-lobar insula and medial frontal gyrus playing a crucial role in the analgesic mechanism for both acute and chronic conditions. In our study on acupuncture treatment for ANSLBP, we identified four clusters of positive activation (right sub-lobar insula, inferior parietal lobule, MFG, and cingulate gyrus) and seven clusters of negative activation (left sub-lobar insula, cingulate gyrus, pulvinar of the thalamus, superior frontal gyrus, right parahippocampal gyrus, MFG, and angular gyrus). In contrast, during our research on CNSLBP, we discovered two clusters of positive activation (right and left sub-lobar insula) and two clusters of negative activation (left precentral gyrus and right MFG). Subgroup analyses revealed different neuroimaging outcomes based on duration. Despite these findings, the quality of evidence and strength of recommendations were rated “very low.” by the GRADE approach, highlighting the need for methodological improvements in fMRI studies on acupuncture for NSLBP.

## Data Availability

The datasets presented in this study can be found in online repositories. The names of the repository/repositories and accession number(s) can be found in the article/Supplementary material.
