# Supplementary material for: Neuroimaging evidence for central mechanisms of acupuncture in non-specific low back pain: a systematic review and meta-analysis
Source: Front Med (Lausanne). 2025 Oct 17;12:1657241. doi: 10.3389/fmed.2025.1657241 (PMC12575243; doi:10.3389/fmed.2025.1657241)

**Supplementary Materials**

**Acupuncture for non-specific low back pain: a meta-analysis of functional magnetic resonance imaging studies**

[**Search strategy 1**](#_Toc178512861)

[**Supplementary Table 1. The results of risk of bias assessment of randomised controlled trials by the Risk of Bias 2. 6**](#_Toc178512862)

[**Supplementary Table 2. The results of risk of bias assessment of non-randomised controlled trials by the Risk Of Bias In Non-randomised Studies-of Interventions. 7**](#_Toc178512863)

[**Supplementary Table 3. Grading of Recommendations, Assessment, Development and Evaluation of acupuncture compared to control group for non-specific low back pain. 8**](#_Toc178512864)

[**Supplementary Table 4. Positive activating clusters of acute non-specific low back pain after acupuncture. 9**](#_Toc178512865)

[**Supplementary Table 5. Negative activating clusters of acute non-specific low back pain after acupuncture. 9**](#_Toc178512866)

[**Supplementary Table 6. Positive activating clusters of chronic non-specific low back pain after acupuncture. 1**](#_Toc178512867)**0**

[**Supplementary Table 7. Negative activating clusters of chronic non-specific low back pain after acupuncture. 10**](#_Toc178512868)

[**Supplementary Table 8. Comparison of brain clusters between acute and chronic non-specific low back pain after acupuncture. 11**](#_Toc178512869)

[**Supplementary Figure 1. Forest plot of the chronic non-specific low back pain. 12**](#_Toc178512936)

# Search strategy

**China National Knowledge Infrastructure (CNKI, Chinese Database) (n=25)**

1. SU=(zhenjiu + zhenci + cijiu + fuzhen + erzhen + dianzhen + meihuazhen + pifuzhen + yaozhenliaofa + wenzhenjiu)
2. SU=(yaotong + yaobeitong + beitong + yaoteng + feiteyixingyaotong)
3. SU=(MRI + fMRI + gongnengcigongzhenchengxiang + gongnengxingcigongzhenchengxiang + xueyangshuipingyilai + shenjingyingxiangxue + gongnengxinglianjie + fMRI jihuo)
4. #1 AND #2 AND #3

**Wanfang Database(WF, Chinese Database) (n=236)**

1. zhenjiu OR zhenci OR cijiu OR fuzhen OR erzhen OR dianzhen OR meihuazhen OR pifuzhen OR yaozhenliaofa OR wenzhenjiu
2. yaotong OR yaobeitong OR beitong OR yaoteng OR feiteyixingyaotong
3. MRI OR fMRI OR gongnengcigongzhenchengxiang OR gongnengxingcigongzhenchengxiang OR xueyangshuipingyilai OR shenjingyingxiangxue OR gongnengxinglianjie OR fMRI jihuo
4. #1 AND #2 AND #3

**Chinese Biomedical Literature Database(CBM, Chinese Database) (n=97)**

1. zhenjiu OR zhenci OR cijiu OR fuzhen OR erzhen OR dianzhen OR meihuazhen OR pifuzhen OR yaozhenliaofa OR wenzhenjiu
2. yaotong OR yaobeitong OR beitong OR yaoteng OR feiteyixingyaotong
3. MRI OR fMRI OR gongnengcigongzhenchengxiang OR gongnengxingcigongzhenchengxiang OR xueyangshuipingyilai OR shenjingyingxiangxue OR gongnengxinglianjie OR fMRI jihuo
4. #1 AND #2 AND #3

**Chongqing VIP Database(VIP, Chinese Database) (n=160)**

1. zhenjiu OR zhenci OR cijiu OR fuzhen OR erzhen OR dianzhen OR meihuazhen OR pifuzhen OR yaozhenliaofa OR wenzhenjiu
2. yaotong OR yaobeitong OR beitong OR yaoteng OR feiteyixingyaotong
3. MRI OR fMRI OR gongnengcigongzhenchengxiang OR gongnengxingcigongzhenchengxiang OR xueyangshuipingyilai OR shenjingyingxiangxue OR gongnengxinglianjie OR fMRI jihuo
4. #1 AND #2 AND #3

**PubMed (n=81)**

1. "Acupuncture[Mesh]" OR "Pharmacopuncture" OR "Acupuncture Analgesia" OR "Analgesia, Acupuncture" OR "Acupuncture Anesthesia" OR "Anesthesia, Acupuncture" OR "Acupuncture therapy" OR "Pharmacoacupuncture Therapy" OR "Acupuncture, Ear" OR "Manual acupuncture" OR Electroacupuncture OR "Skin acupuncture" OR "Warm acupuncture" OR "Acupotom*" OR "Abdominal Acupuncture" OR "needling and moxibustion" OR "acupuncture and moxibustion" OR "percussopunctator" OR "pyonex" OR "plum blossom needle"
2. "Low Back Pain"[Mesh] OR "Back Pain*, Low" OR "Low Back Pain*" OR "Pain*, Low Back" OR "Ache*, Low Back" OR "Back Ache*, Low" OR "Low Back Ache*" OR "Backache*, Low" OR "Low Backache*" OR "Back Pain*, Lower" OR "Lower Back Pain*" OR "Pain*, Lower Back" OR "Lumbago" OR "Low Back Pain, Mechanical" OR "Mechanical Low Back Pain" OR "Low Back Pain, Posterior Compartment" OR "Low Back Pain, Postural" OR "Postural Low Back Pain" OR "Low Back Pain, Recurrent" OR "Recurrent Low Back Pain" OR backache OR "back-ache" OR "back disorder"[Title/Abstract] OR "Non-specific low back pain"
3. "Magnetic Resonance Imaging"[Mesh] OR "Imaging, Magnetic Resonance" OR "NMR Imaging" OR "Imaging, NMR" OR "Zeugmatography" OR "Tomography, MR" OR "Steady-State Free Precession MRI" OR "Steady State Free Precession MRI" OR "NMR Tomography" OR "Tomography, NMR" OR "MR Tomography" OR "Tomography, Proton Spin" OR "Proton Spin Tomography" OR "Magnetization Transfer Contrast Imaging" OR "fMRI" OR "Magnetic Resonance Imaging, Functional" OR "Functional MRI*" OR "MRIs, Functional" OR "MRI, Functional" OR "Functional Magnetic Resonance Imaging" OR "MRI Scan*" OR "Scan*, MRI" OR "Chemical Shift Imaging*" OR "Imaging*, Chemical Shift" OR "Shift Imaging*, Chemical" OR "Echo Imaging*, Spin" OR "Imaging*, Spin Echo" OR "Spin Echo Imaging*" OR "Image, Magnetic Resonance" OR "Magnetic Resonance Image*" OR "Resonance Image, Magnetic"
4. "Neuroimaging"[Mesh] OR "Brain Imaging" OR "Imaging, Brain"
5. "Functional Neuroimaging"[Mesh] OR "Neuroimaging, Functional" OR "Brain Imaging*, Functional" OR "Functional Brain Imaging*" OR "Imaging*, Functional Brain"
6. #3 OR #4 OR #5
7. #1 AND #2 AND #6

**Web of science (n=130)**

1. TS= ("Acupuncture" OR "Pharmacopuncture" OR "Acupuncture Analgesia" OR "Analgesia, Acupuncture" OR "Acupuncture Anesthesia" OR "Anesthesia, Acupuncture" OR "Acupuncture therapy" OR "Pharmacoacupuncture Therapy" OR "Acupuncture, Ear" OR "Manual acupuncture" OR Electroacupuncture OR "Skin acupuncture" OR "Warm acupuncture" OR "Acupotomy" OR "Acupotomies" OR "Abdominal Acupuncture" OR "needling and moxibustion" OR "acupuncture and moxibustion" OR "percussopunctator" OR "pyonex" OR "plum-blossom needle" OR "plum blossom needles")
2. TS=("Back Pain*, Low" OR "Low Back Pain*" OR "Pain*, Low Back" OR "Ache*, Low Back" OR "Back Ache*, Low" OR "Low Back Ache*" OR "Backache*, Low" OR "Low Backache*" OR "Back Pain*, Lower" OR "Lower Back Pain*" OR "Pain*, Lower Back" OR "Lumbago" OR "Low Back Pain, Mechanical" OR "Mechanical Low Back Pain" OR "Low Back Pain, Posterior Compartment" OR "Low Back Pain, Postural" OR "Postural Low Back Pain" OR "Low Back Pain, Recurrent" OR "Recurrent Low Back Pain" OR backache OR "back-ache" OR "back disorder" OR "Non-specific low back pain")
3. TS="Magnetic Resonance Imaging"[Mesh] OR "Imaging, Magnetic Resonance" OR "NMR Imaging" OR "Imaging, NMR" OR "Zeugmatography" OR "Tomography, MR" OR "Steady-State Free Precession MRI" OR "Steady State Free Precession MRI" OR "NMR Tomography" OR "Tomography, NMR" OR "MR Tomography" OR "Tomography, Proton Spin" OR "Proton Spin Tomography" OR "Magnetization Transfer Contrast Imaging" OR "fMRI" OR "Magnetic Resonance Imaging, Functional" OR "Functional MRI*" OR "MRIs, Functional" OR "MRI, Functional" OR "Functional Magnetic Resonance Imaging" OR "MRI Scan*" OR "Scan*, MRI" OR "Chemical Shift Imaging*" OR "Imaging*, Chemical Shift" OR "Shift Imaging*, Chemical" OR "Echo Imaging*, Spin" OR "Imaging*, Spin Echo" OR "Spin Echo Imaging*" OR "Image, Magnetic Resonance" OR "Magnetic Resonance Image*" OR "Resonance Image, Magnetic" OR "Neuroimaging" OR "Brain Imaging" OR "Imaging, Brain" OR "Functional Neuroimaging" OR "Neuroimaging, Functional" OR "Neuroimaging, Functional" OR "Brain Imaging*, Functional" OR "Functional Brain Imaging*" OR "Imaging*, Functional Brain")
4. #1 AND #2 AND #3

**Embase (n=348)**

1. 'Acupuncture'/exp OR 'Acupuncture therapy' OR 'shonishin'
2. 'auricular acupuncture'/exp OR 'acupuncture, ear' OR 'acupuncture, earlobe' OR 'auriculo-acupuncture' OR 'auriculoacupuncture' OR 'auriculotherapy' OR 'ear acupuncture' OR 'earlobe acupuncture' OR 'auricular acupuncture'
3. 'electroacupuncture'/exp OR 'acupuncture, electric' OR 'electric acupuncture' OR 'electrical acupoint stimulation' OR 'electrical acupuncture' OR 'electro-acupuncture' OR 'electrode acupuncture' OR 'electronic acupuncture' OR 'electroacupuncture'
4. 'pharmacopuncture'/exp OR 'herb acupuncture' OR 'herbal acupuncture' OR 'herbalized acupuncture' OR 'pharmaco-acupuncture' OR 'pharmaco-puncture' OR 'pharmacoacupuncture' OR 'pharmacopuncture'
5. 'warm acupuncture'/exp OR 'burnt needle therapy' OR 'fire acupuncture' OR 'fire needle acupuncture' OR 'fire needle therapy' OR 'fire needling' OR 'heat acupuncture' OR 'heat-type acupuncture' OR 'thermal acupuncture' OR 'thermo-acupuncture' OR 'thermoacupuncture' OR 'warm needle acupuncture' OR 'warm acupuncture'
6. 'Skin acupuncture' OR 'Abdominal Acupuncture' OR 'acupuncture and moxibustion' OR percussopunctator OR pyonex OR 'plum-blossom needle' OR 'plum blossom needles'
7. #1 OR #2 OR #3 OR #4 OR #5 OR #6
8. 'low back pain'/exp OR 'acute low back pain' OR 'back pain, low' OR 'chronic low back pain' OR 'loin pain' OR 'low backache' OR 'low backpain' OR 'lowback pain' OR 'lower back pain' OR 'lumbago' OR 'lumbal pain' OR 'lumbal syndrome' OR 'lumbalgesia' OR 'lumbalgia' OR 'lumbar pain' OR 'lumbar spine syndrome' OR 'lumbodynia' OR 'lumbosacral pain' OR 'lumbosacral root syndrome' OR 'lumbosacroiliac strain' OR 'pain, low back' OR 'pain, lumbosacral' OR 'strain, lumbosacroiliac' OR 'low back pain'
9. 'nuclear magnetic resonance imaging'/exp OR 'imaging, magnetization transfer' OR 'magnetic resonance imaging' OR 'magnetic resonance tomography' OR 'magnetization transfer imaging' OR 'mr imaging' OR 'MRI' OR 'NMR imaging' OR 'nuclear magnetic resonance imaging'
10. 'functional magnetic resonance imaging'/exp OR 'fMRI' OR 'functional MRI' OR 'magnetic resonance imaging, functional' OR 'R-fMRI' OR 'resting state functional magnetic resonance imaging' OR 'rsfMRI' OR 'functional magnetic resonance imaging'
11. 'neuroimaging'/exp OR 'neural imaging' OR 'neuro-imaging' OR 'neuroimaging'
12. #9 OR #10 OR #11
13. #7 AND #8 AND #12

**Cochrane Library (n=45)**

1. MeSH descriptor: [Acupuncture] explode all trees
2. ('Pharmacopuncture' OR 'Acupuncture Analgesia' OR 'Analgesia, Acupuncture' OR 'Acupuncture Anesthesia' OR 'Anesthesia, Acupuncture' OR 'Acupuncture therapy' OR 'Pharmacoacupuncture Therapy' OR 'Acupuncture, Ear' OR 'Manual acupuncture' OR Electroacupuncture OR 'Skin acupuncture' OR 'Warm acupuncture' OR 'Acupotom*' OR 'Abdominal Acupuncture' OR 'needling and moxibustion' OR 'acupuncture and moxibustion' OR 'percussopunctator' OR 'pyonex' OR 'plum blossom needle*'):ti,ab,kw
3. #1 OR #2
4. MeSH descriptor: [Low Back Pain] explode all trees
5. ('Back Pain*, Low' OR 'Low Back Pain*' OR 'Pain*, Low Back' OR 'Ache*, Low Back' OR 'Back Ache*, Low' OR 'Low Back Ache*' OR 'Backache*, Low' OR 'Low Backache*' OR 'Back Pain*, Lower' OR 'Lower Back Pain*' OR 'Pain*, Lower Back' OR 'Lumbago' OR 'Low Back Pain, Mechanical' OR 'Mechanical Low Back Pain' OR 'Low Back Pain, Posterior Compartment' OR 'Low Back Pain, Postural' OR 'Postural Low Back Pain' OR 'Low Back Pain, Recurrent' OR 'Recurrent Low Back Pain'):ti,ab,kw
6. #4 OR #5
7. MeSH descriptor: [Magnetic Resonance Imaging] explode all trees
8. MeSH descriptor: [Neuroimaging] explode all trees
9. MeSH descriptor: [Functional Neuroimaging] explode all trees
10. ('Imaging, Magnetic Resonance' OR 'NMR Imaging' OR 'Imaging, NMR' OR 'Zeugmatography' OR 'Tomography, MR' OR 'Steady-State Free Precession MRI' OR 'Steady State Free Precession MRI' OR 'NMR Tomography' OR 'Tomography, NMR' OR 'MR Tomography' OR 'Tomography, Proton Spin' OR 'Proton Spin Tomography' OR 'Magnetization Transfer Contrast Imaging' OR 'fMRI' OR 'Magnetic Resonance Imaging, Functional' OR 'Functional MRI*' OR 'MRIs, Functional' OR 'MRI, Functional' OR 'Functional Magnetic Resonance Imaging' OR 'MRI Scan*' OR 'Scan*, MRI' OR 'Chemical Shift Imaging*' OR 'Imaging*, Chemical Shift' OR 'Shift Imaging*, Chemical' OR 'Echo Imaging*, Spin' OR 'Imaging*, Spin Echo' OR 'Spin Echo Imaging*' OR 'Image, Magnetic Resonance' OR 'Magnetic Resonance Image*' OR 'Resonance Image, Magnetic' OR 'Brain Imaging' OR 'Imaging, Brain' OR 'Neuroimaging, Functional' OR 'Brain Imaging*, Functional' OR 'Functional Brain Imaging*' OR 'Imaging*, Functional Brain'):ti,ab,kw
11. #7 OR #8 OR #9 OR #10
12. #3 AND #6 AND #11

# Supplementary Table 1. The results of risk of bias assessment of randomised controlled trials by the Risk of Bias 2.

| **Author (year)** | **Bias arising from the randomization process** | **Bias due to deviations from intended interventions** | **Bias due to missing outcome data** | **Bias in measurement of the outcome** | **Bias in selection of the reported result** | **Overall risk of bias** |
| --- | --- | --- | --- | --- | --- | --- |
| Shi  (2015) | Some concerns | Low | Low | Low | Low | Some concerns |
| Liu (2013) | Some concerns | Low | Low | Low | Low | Some concerns |
| Liu (2013) | Some concerns | Low | Low | Low | Low | Some concerns |
| Yu (2020) | Low | Low | Low | Low | Low | Low |
| Tu  (2019) | Some concerns | Low | Low | Low | Low | Some concerns |
| Kim (2020) | Some concerns | Low | Low | Low | Low | Some concerns |
| Makary (2018) | Some concerns | Low | Low | Low | Low | Some concerns |
| Lee (2019) | Some concerns | Low | Low | Low | Low | Some concerns |

#

# Supplementary Table 2. The results of risk of bias assessment of non-randomised controlled trials by the Risk Of Bias In Non-randomised Studies-of Interventions.

| **Author (year)** | **Bias due to confounding** | **Bias in selection of participants into study** | **Bias in classification of interventions** | **Bias due to deviations from intended interventions** | **Bias due to missing data** | **Bias in measurement of outcomes** | **Bias in selection of the reported result** | **Overall risk of bias** |
| --- | --- | --- | --- | --- | --- | --- | --- | --- |
| Xiang (2019) | Low | Low | Low | Low | Low | Low | Low | Low |
| Jia (2021) | Low | Low | Low | Low | Low | Low | Low | Low |

# Supplementary Table 3. Grading of Recommendations, Assessment, Development and Evaluation of acupuncture compared to control group for non-specific low back pain.

| **Acupuncture compared to control group for non-specific low back pain** | | | | | | |
| --- | --- | --- | --- | --- | --- | --- |
| **Patient or population:** patients with non-specific low back pain  **Settings:**  **Intervention:** Acupuncture  **Comparison:** control group | | | | | | |
| **Outcomes** | **Illustrative comparative risks* (95% CI)** | | **Relative effect**  **(95% CI)** | **No of Participants**  **(studies)** | **Quality of the evidence**  **(GRADE)** | **Comments** |
|  | Assumed risk | Corresponding risk |  |  |  |  |
|  | **Control group** | **Acupuncture** |  |  |  |  |
| **Vas**  Vas | The mean vas in the control groups was  **1** | The mean vas in the intervention groups was  **0.78 lower**  (0 higher to 0.31 lower) |  | 358  (5 studies) | ⊕⊝⊝⊝  **very low**^1,2^ |  |
| *The basis for the **assumed risk** (e.g. the median control group risk across studies) is provided in footnotes. The **corresponding risk** (and its 95% confidence interval) is based on the assumed risk in the comparison group and the **relative effect** of the intervention (and its 95% CI).  **CI:** Confidence interval; | | | | | | |
| GRADE Working Group grades of evidence  **High quality:** Further research is very unlikely to change our confidence in the estimate of effect.  **Moderate quality:** Further research is likely to have an important impact on our confidence in the estimate of effect and may change the estimate.  **Low quality:** Further research is very likely to have an important impact on our confidence in the estimate of effect and is likely to change the estimate.  **Very low quality:** We are very uncertain about the estimate. | | | | | | |
| ^1^ The lack of allocation concealment, lack of blinding, failure to adhere to an analysis according to intertion-to-treat principle.  ^2^ All studies with limited sample size included. | | | | | | |

**Supplementary Table 4.** Positive activating clusters of acute non-specific low back pain after acupuncture.

| Cluster # | Side | Anatomical Location | Peak MNI coordinate | | | ALE value (×10^-2^) | *P* value(×10^-8^) | Z value |
| --- | --- | --- | --- | --- | --- | --- | --- | --- |
|  |  |  | X | Y | Z |  |  |  |
| 1 | R | Sub-lobar.Insula.Gray Matter.Brodmann area 13. | 50 | 6 | 12 | 0.217 | 9.719 | 5.205 |
| 2 | R | Parietal Lobe.Inferior Parietal Lobule.Gray Matter.Brodmann area 40. | 62 | -26 | 34 | 0.229 | 3.576 | 5.387 |
| 3 | R | Frontal Lobe.Medial Frontal Gyrus.Gray Matter.Brodmann area 6 | 12 | 0 | 60 | 0.191 | 61.758 | 4.850 |
| 4 | R | Limbic Lobe.Cingulate Gyrus.Gray Matter.Brodmann area 31. | 18 | -24 | 39 | 0.190 | 66.287 | 4.850 |

**Supplementary Table 5.** Negative activating clusters of acute non-specific low back pain after acupuncture.

| Cluster # | Side | Anatomical Location | Peak MNI coordinate | | | ALE value (×10^-2^) | *P* value (×10^-8^) | Z value |
| --- | --- | --- | --- | --- | --- | --- | --- | --- |
|  |  |  | X | Y | Z |  |  |  |
| 1 | L | Sub-lobar.Insula.Gray Matter.Brodmann area 13. | -41 | -13 | 15 | 0.274 | ＜0.001 | 6.363 |
| 2 | L | Limbic Lobe.Cingulate Gyrus.Gray Matter.Brodmann area 32. | 0 | 33 | 21 | 0.282 | ＜0.001 | 7.026 |
| 3 | L | Sub-lobar.Thalamus.Gray Matter.Pulvinar. | -4 | -30 | -2 | 0.188 | 228.940 | 4.583 |
| 4 | R | Limbic Lobe.Parahippocampal Gyrus.Gray Matter.Brodmann area 35. | 24 | -27 | -18 | 0.190 | 198.221 | 4.613 |
| 5 | R | Frontal Lobe.Medial Frontal Gyrus.Gray Matter.Brodmann area 8. | 14 | 33 | 44 | 0.187 | 248.138 | 4.566 |
| 6 | R | Parietal Lobe.Angular Gyrus.Gray Matter.Brodmann area 39. | 54 | -60 | 39 | 0.190 | 198.222 | 4.613 |
| 7 | L | Frontal Lobe.Superior Frontal Gyrus.Gray Matter.Brodmann area 6 | -14 | 34 | 52 | 0.187 | 248.138 | 4.566 |

**Supplementary Table 6.** Positive activating clusters of chronic non-specific low back pain after acupuncture.

| Cluster # | Side | Anatomical Location | Peak MNI coordinate | | | ALE value (×10^-2^) | *P* value (×10^-8^) | Z value |
| --- | --- | --- | --- | --- | --- | --- | --- | --- |
|  |  |  | X | Y | Z |  |  |  |
| 1 | R | Sub-lobar.Insula.Gray Matter.Brodmann area 13 | 46 | -2 | 2 | 0.185 | 163.861 | 4.653 |
| 2 | L | Sub-lobar.Insula.Gray Matter.Brodmann area 13 | -42 | -16 | 2 | 0.155 | 4236.508 | 3.931 |

**Supplementary Table 7.** Negative activating clusters of chronic non-specific low back pain after acupuncture.

| Cluster # | Side | Anatomical Location | Peak MNI coordinate | | | ALE value(×10^-2^) | *P* value (×10^-8^) | Z value |
| --- | --- | --- | --- | --- | --- | --- | --- | --- |
|  |  |  | X | Y | Z |  |  |  |
| 1 | L | Frontal Lobe.Precentral Gyrus.Gray Matter.Brodmann area 44 | -56 | 12 | 6 | 0.0949 | 32523.14 | 3.410 |
| 2 | R | Frontal Lobe.Middle Frontal Gyrus.Gray Matter.Brodmann area 8 | 33 | 40 | 42 | 0.0949 | 32523.14 | 3.410 |

**Supplementary Table 8.** Comparison of brain clusters between acute and chronic non-specific low back pain after acupuncture.

| Non-specific low back pain | Activating | Cluster # | Side | Anatomical Location |
| --- | --- | --- | --- | --- |
| Acute non-specific low back pain | Positive | 1 | R | Sub-lobar.Insula.Gray Matter.Brodmann area 13 |
|  | Positive | 2 | R | Parietal Lobe.Inferior Parietal Lobule.Gray Matter.Brodmann area 40. |
|  | Positive | 3 | R | Frontal Lobe.Medial Frontal Gyrus.Gray Matter.Brodmann area 6 |
|  | Positive | 4 | R | Limbic Lobe.Cingulate Gyrus.Gray Matter.Brodmann area 31. |
|  | Negative | 1 | L | Sub-lobar.Insula.Gray Matter.Brodmann area 13. |
|  | Negative | 2 | L | Limbic Lobe.Cingulate Gyrus.Gray Matter.Brodmann area 32. |
|  | Negative | 3 | L | Sub-lobar.Thalamus.Gray Matter.Pulvinar. |
|  | Negative | 4 | R | Limbic Lobe.Parahippocampal Gyrus.Gray Matter.Brodmann area 35. |
|  | Negative | 5 | R | Frontal Lobe.Medial Frontal Gyrus.Gray Matter.Brodmann area 8. |
|  | Negative | 6 | R | Parietal Lobe.Angular Gyrus.Gray Matter.Brodmann area 39. |
|  | Negative | 7 | L | Frontal Lobe.Superior Frontal Gyrus.Gray Matter.Brodmann area 6 |
| Chronic non-specific low back pain | Positive | 1 | R | Frontal Lobe.Precentral Gyrus.Gray Matter.Brodmann area 44 |
|  | Positive | 2 | L | Frontal Lobe.Middle Frontal Gyrus.Gray Matter.Brodmann area 8 |
|  | Negative | 1 | L | Frontal Lobe.Precentral Gyrus.Gray Matter.Brodmann area 44 |
|  | Negative | 2 | R | Frontal Lobe.Middle Frontal Gyrus.Gray Matter.Brodmann area 8 |

**Supplementary Figure 1.** Forest plot of the chronic non-specific low back pain.


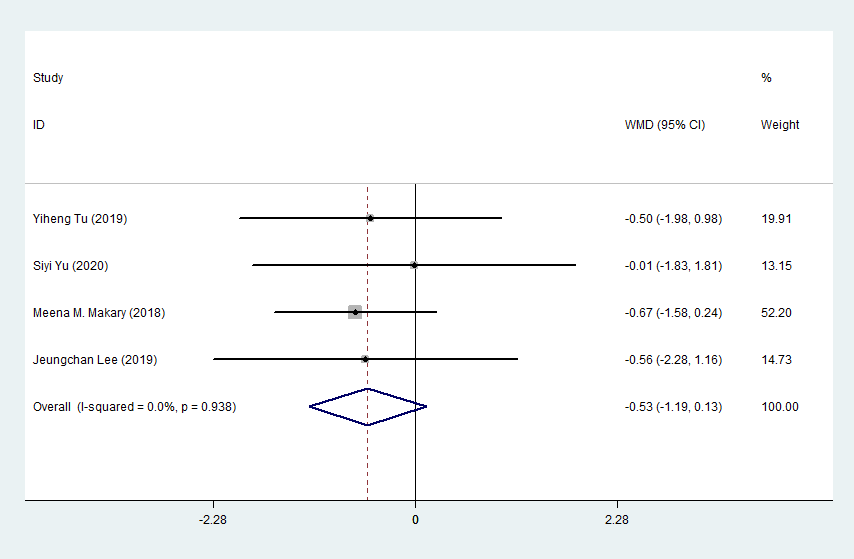


**Supplementary Figure 2.** Top 10 brain areas of MT-related alterations of included neuroimaging studies.


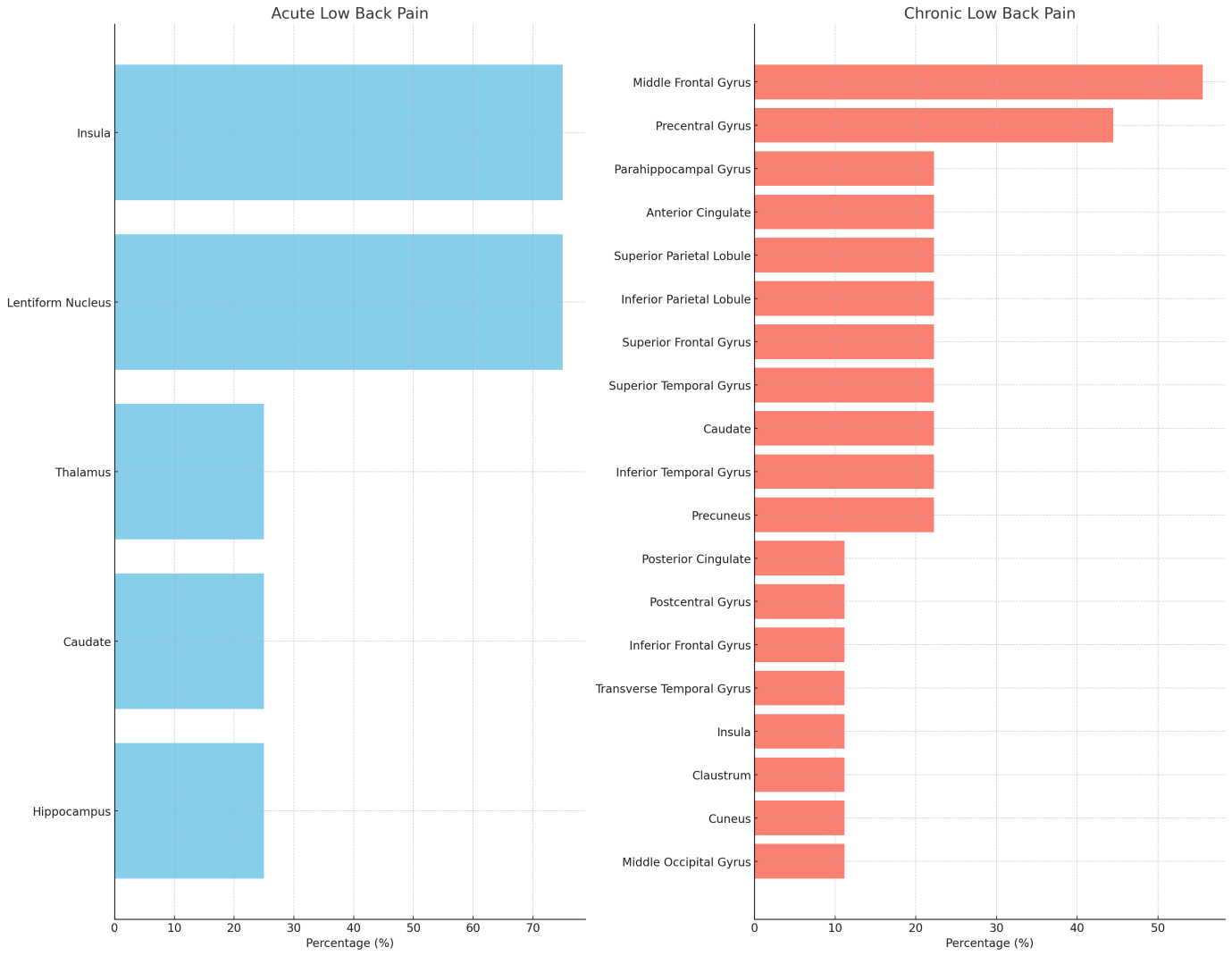

Supplement: Supplementary file 1 [file Data_Sheet_1.docx]
